# An efficient and adaptive data-hiding scheme based on secure random matrix

**DOI:** 10.1371/journal.pone.0222892

**Published:** 2019-10-02

**Authors:** Mingwu Zhang, Shaochen Zhang, Lein Harn

**Affiliations:** 1 School of Computer Sciences, Hubei University of Technology, Wuhan, Hubei Province, China; 2 Hubei Key Laboratory of Intelligent Geo-Information Processing, China University of Geosciences, Wuhan, Hubei Province, China; 3 Department of Computer Science Electrical Engineering, University of Missouri-Kansas City, Kansas City, State of Missouri, United States of America; Victoria University, AUSTRALIA

## Abstract

There is a vivid research on securely deliver a secret message by using a data hiding technique in digital images. However, most existing solutions of data-hiding need to encrypt secret data first, and embed the encrypt message into cover image, which is not promising any security once the embedding algorithm is leaked. In this paper, an efficient and adaptive data hiding scheme is presented which is based on a new reference matrix named secure reference matrix. The scheme is flexible enough to meet different data hiding capacities and image quality as needed, and we allow the reference matrix to be more secure since it is randomly generated each time and make it much harder for being attack with exhaustive manner by extending the possible solution. We give the detail protocol for our scheme and provide the practical test and performance analysis. Experimental results show that our scheme has a better flexibility and efficiency in embedding process, and the average value of Peak Signal to Noise Ratio (PSNR) for the stego-images which are generated by our scheme have a higher visual quality for different embedding capacities, and our scheme is more efficient compared with related work.

## Introduction

With the vivid steganography application of data hiding in open images, steganography of digital image is a very useful tool for secure transmission of secret data, since it’s very hard for human eyes to distinguish slightly modified stego image from a cover image. The steganography technique changes few bits in the original image and encodes a message into it. Unlike traditional encryption mechanism [1, 2], in which a message is being hidden in the corresponding ciphertext [3], steganography hides data in plain view, inside a file such as a picture. And the security for the steganography is based on encrypting the secret message [4] or scrambling embedded locations before embedding into cover image to ensure the security of secret information, which is making the embedding process complex and taking extra time.

In order to evaluate the performance of a data hiding scheme in the steganography model, two important parameters are considered, namely *embedded capacity* and *visual quality*[5, 6]. Actually, these two parameters are inversely proportional to each other: the more embedded secret information, the more noise existed in the image, and thus the image quality will inevitably decline. We should guarantee that the image that has embedded the hiding data will look like just a normal and innocent image. A good data-hiding method should be capable of evading visual and statistical detection [4] while providing an adjustable payload [7]

In 2006, Mielikainen [8] introduced the LSB matching revisited scheme that embedding two bits into a pixel pair of cover image. Zhang and Wang [9] proposed the *exploiting modification direction* (EMD) data hiding scheme, to overcome the weakness in [8], but the payload only 1.161 bpp for embedding a base-5 numeral system into a pixel pair. In [9], each secret digit in a base-(2*n* + 1) numeral system was carried by a pixel-group (*n* pixels) and at most one pixel of the pixel-group must be changed, which achieves a higher embedding capacity and provides better stego-image quality than previous approaches. Kim et al. [10] provided a further step to improve the quality of the Mielikainen’s method [8].

In 2008, Chang, Chou and Kieu [11] presented a Sudoku-based data hiding scheme to improve the security, a secret digit in *base* − 9 numeral system is embedded into each pair of pixels, but their scheme had a large search set and only could embed *base* − 9 numeral system, not flexible enough to multiple embedded requirements. In 2012, Hong and Chen [7] proposed an adaptive pixel pair matching (APPM) method, two pixels are scanned as an embedding unit and designed neighbourhood set is specially employed to embed secret message digits, but they only scrambling embedding position for the security, the scrambled plain data can be extracted without key or additional information. In 2014, J. Chen [12] proposed a pixel pair matching(PPM) with Pixel value difference(PVD) method making the secret data embedded adaptively into pixel pair using two reference tables and had the resistance to chi-square steganalysis. And Chang, Liu and Nguyen [13] used a novel matrix, namely *turtle shell matrix* in this year. Their scheme increased the embedding capacity and thus provides higher efficiency by embedding a digit in *base* − 8 means 2 bits into each pixel pair through the turtle shell matrix increasing the embedding efficiency. And make flexible embedding version in 2017 [14], and the faster search version and in 2018 [6]. But once the embedding algorithm is revealed for the stego image, any malicious individual could extract the secret information easily, so encrypting the secret data [15] is a necessary for their scheme. Keeping this in mind several schemes based on secret keys have been proposed in recent years [16]. Recently, Yadav and Ojha [17] proposed a more efficient scheme to embed the secret data into random locations of the image through chaotic systems for security, which provides a higher payload and imperceptibility.

Inspired by the idea of Sudoku technique [11], in this work, we construct a novel reference matrix, namely *secure random matrix* (SRM), to guide the modification of cover pixels and obtain better visual quality and increase the security level in image-based data hiding. Our contributions are described as follows.

1. Based on Chang et al.’s scheme [11], We design a new secure random matrix to make it more efficient in embedding process and improve the visual quality of stego images.
2. Based on the technique of secure random matrix and pseudo-random generators, we propose a more flexible scheme, which could hide different sizes of data adaptively to meet different needs.
3. we give the experimental analysis for the practical image-based data hiding, and also provide the analysis of improvement the security of the steganography. Experimental results show that the average value of our proposed scheme’s peak signal-to-noise ratio obtains a better visual quality in different embedding capacity compared with the other schemes, and has higher efficiency in data hiding process.

The rest of this paper is organized as follows. Section 2 describes the design concept of our scheme. Section 3 presents a detail scheme of our proposed secure random matrix, and also explore the algorithms of our data embedding and extracting. In Section 4, it provides the experimental results and the performance analysis, and also gives the comparison with related works. Finally, the conclusion of this paper is drawn in Section 5.

## Design concept

In order to improve the utilization of cover image redundancy space, we use the inherent properties of the pseudo-random number [18] generator to improve the embedding efficiency with the reference matrix. Our design philosophy is based on the following theorem.

**Theorem 1**. *For any b* × *b matrix M*_1_ *containing non-overlapping* 0 − (*b*^2^ − 1) *numbers, scrambles the values of each column in order and splices them on the right side of the matrix M*_1_ *to form a b* × 256 *matrix M*_2_. *Then take any number of the middle row of the matrix M*_2_, *the b* × 3 *matrix centered on this number is also a matrix containing non-overlapping* 0-(*b*^2^ − 1) *digits*.

*Proof*. Suppose that we sequentially scramble and splicing each column, we arbitrarily select a number in the second row of the matrix *M*_2_ to generate the matrix *M*_*a*_ with this number as the center of the matrix. The three columns of the *b* × *b* matrix *M*_*a*_ are sequentially scrambled, and since the numbers included in the three columns of *M*_1_ do not overlap, the numbers included in the scrambled matrix *M*_*a*_ do not overlap (matrix a contains non-overlapping columns of the original matrix *M*_1_)). And due to the matrix a contains only 0-(*b*^2^ − 1) (the scramble does not make the result of the scrambling beyond the range of the original number, just change the order), then select a number of the *b* × 256 matrix *M*_2_ second line to the *b* × *b* matrix whose number is the center of the matrix is also a matrix containing numbers that do not overlap 0-(*b*^2^ − 1) digits.

**Theorem 2**. *Copy the b* × 256 *matrix M*_2_ *generated by Theorem 1 and splice it over the matrix M*_2_ *to obtain a* 256 × 256 *matrix M*_3_. *For any number in the matrix M*_3_ *and the b* × *b matrix centered on this number is also a matrix containing numbers of do not overlap* 0-(*b*^2^ − 1) *digits*.

*Proof*. Suppose we have a number *z* in the optional matrix, and a *b* × *b* matrix *M*_*a*_ with the number *z* as the centre of the matrix. The matrix *M*_*a*_ can be seen as a result obtained by sequentially scrambling the matrix *M*_1_1 which is generated by row swapping of the original matrix *M*_1_. The numbers contained in the scrambled matrix *M*_*a*_ do not overlap (matrix a contains non-overlapping columns of the original matrix *M*_1_), and the matrix *M*_*a*_ contains only 0-8 digits, then a number *z* in an optional matrix (a number that does not include the most edge of the matrix). The *b* × *b* matrix centered at this number *z* is also a matrix containing non-repeating 0-(*b*^2^ − 1) digits.

**Theorem 3**. *There are at least* 10^78^ *possible solutions for the matrix M*_3_ *constructed according to Theorem 1 and Theorem 2*.

*Proof*. According to Theorem 1, the possible solutions to the initial matrix *M*_1_ are calculated as follows:

$$\begin{array}{c} P_{1}=A_{b^{2}}^{b^{2}} \end{array}$$ (1)

And according to Theorem 1, the possible solutions to the matrix *M*_2_ are calculated as follows:

$$\begin{array}{c} P_{2}=\prod_{1}^{256-b}A_{b}^{b} \end{array}$$ (2)

According to the Theorem 2, since the generation of the matrix *M*_3_ is made up of the matrix *M*_2_ copy and splicing, once the matrix *M*_2_ is determined, the matrix *M*_3_ is determined. Therefore, the total possible solutions for the generated 256 × 256 matrix *M*_3_ are calculated as follows:

$$\begin{array}{c} P_{3}=P_{1}\times P_{2}=A_{b^{2}}^{b^{2}}\times \prod_{1}^{253}A_{b^{2}}^{b^{2}} \end{array}$$ (3)

The number of possible solutions is based on the scrambling the numbers of the matrix *M*_1_ and *M*_2_, the least possible solutions will for the case of *b* = 2, because there are least number is scrambled in this case, ie

$$\begin{array}{c} P_{3}=P_{1}\times P_{2}=A_{b^{2}}^{b^{2}}\times \prod_{1}^{253}A_{b^{2}}^{b^{2}}\approx 2\times 10^{78} \end{array}$$ (4)

## Proposed scheme

**Fig 1:**
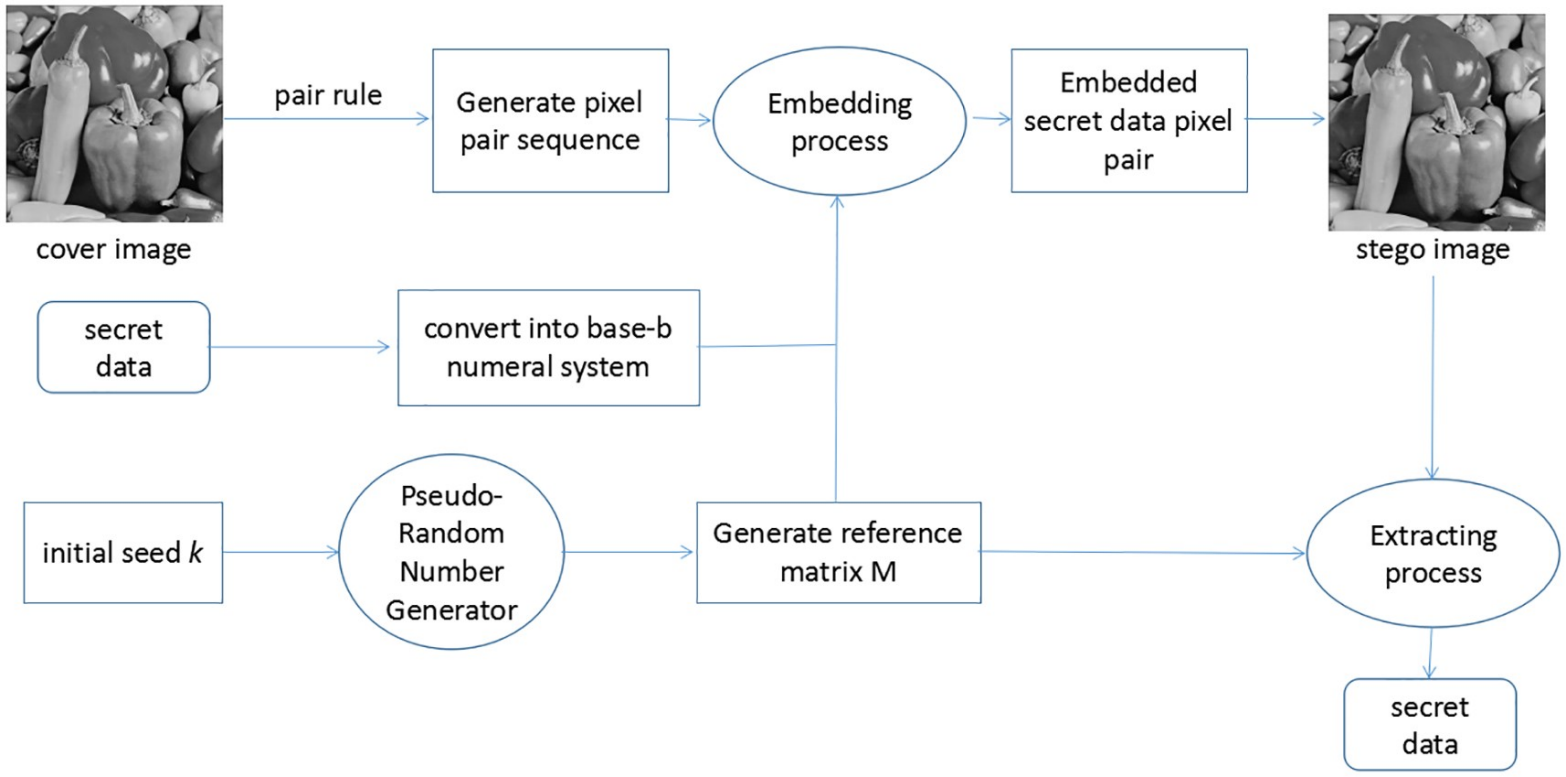
Total construct of the proposed scheme.

In this section, we propose an efficient data hiding scheme, as shown in the Fig 1, which is based on the concept of the secure random matrix. In order to combine the ensure the security of embedding process, the reference matrix is generated by a Pseudo-Random Number Generator (PRNG). The total process of the proposed method is described as follows:

- **Key Generation**: Random select a number *k* as PRNG initial seed required(with length *log*_2_ *k* = λ).
- **Construction of Secure Random Matrix**: Construct a secure random matrix as a reference matrix before the embedding process.
- **Embedding Process**: Pixels in the cover image are paired into pixel pairs, and the secret bit stream is converted into secret digits *d*_*i*_ with the base-9 numeral system, where *i* ∈ (1, 2, 3, ⋯, *N*). Then embedding *d*_*i*_ into the pixel pair according to pre-produced reference matrix.
- **Extracting Process**: With the key *k* as the initial seed of the PRNG, the reference matrix is generated. Then according to the stego image, extract the embedded digits with the inverse process of the embedding process. Finally, retrieve the original secret data from the extracted digits. secret digits S.

### Construction of secure random matrix

The main idea of this paper is to randomize the reference matrix but do not change the characteristics of the matrix because of the randomization. Then embed the value of the secret data into the pixel pair through a secure random matrix. The secure random matrix can satisfy such a requirement. Although we randomly select a number from it, the selected number and the 3 × 3 matrix of eight numbers around it must contain all the numbers in 0-8.

The procedure for constructing the matrix is shown as follows:

Step 1 Generate a sequence of random numbers R through a PRNG, making the initial seed of the PRNG as the secret key for extracting. Then make the value of *R* ranges form 0-*b*^2^ − 1 by the formula: *R*′ = *R* mod *b*^2^.
Step 2 Generate a *b* × *b* initial matrix *M*_1_ by ordering the first nine different numbers of random number sequences *R*′.
Step 3 Scramble the first column of the initial matrix *m* with an unused number of the random number sequence *R*′, and splice the scrambled column with the initial matrix *m* to form a new matrix *m*′.
Step 4 The next column of the initial matrix *m* is scrambled with an unused number of the random number sequence *R*′, and splice the scrambled column with *m*′ formed in the last step to get matrix *m*″.
Step 5 The third column of the initial matrix *m* is scrambled using the same scrambling method as in step 3, and splice the scrambled column with *m*″ formed in the last step to get matrix *m*‴.
Step 6 Repeat steps 3-5 until a *b*×256 matrix *M*′ is formed.
Step 7 Repeat and copy the matrix *M*′ multiple times and splicing them up to form a random security 256×256 matrix *M*.

**Fig 2:**
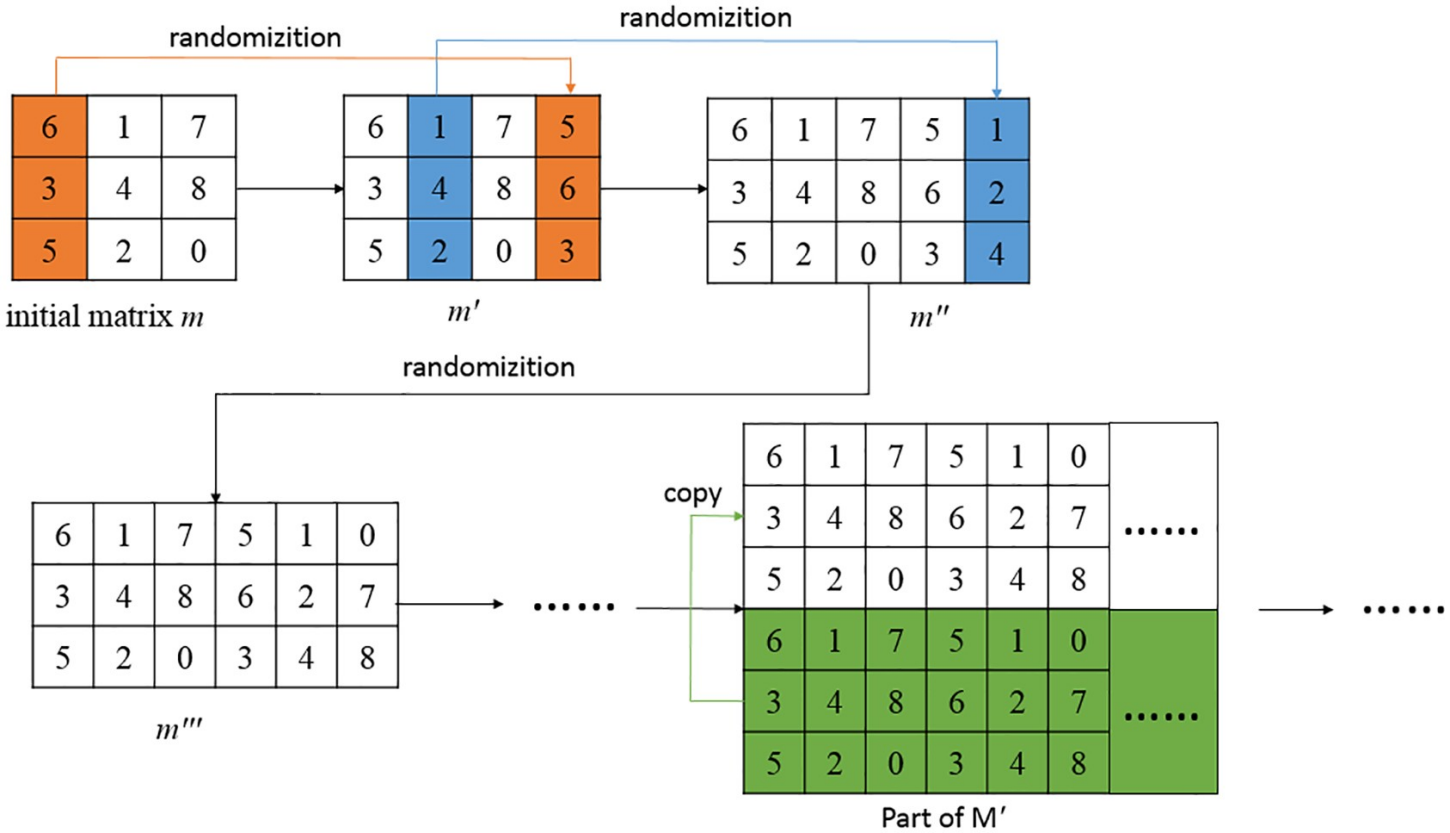
Example of constructing a secure random matrix for *b* = 3.

**Fig 3:**
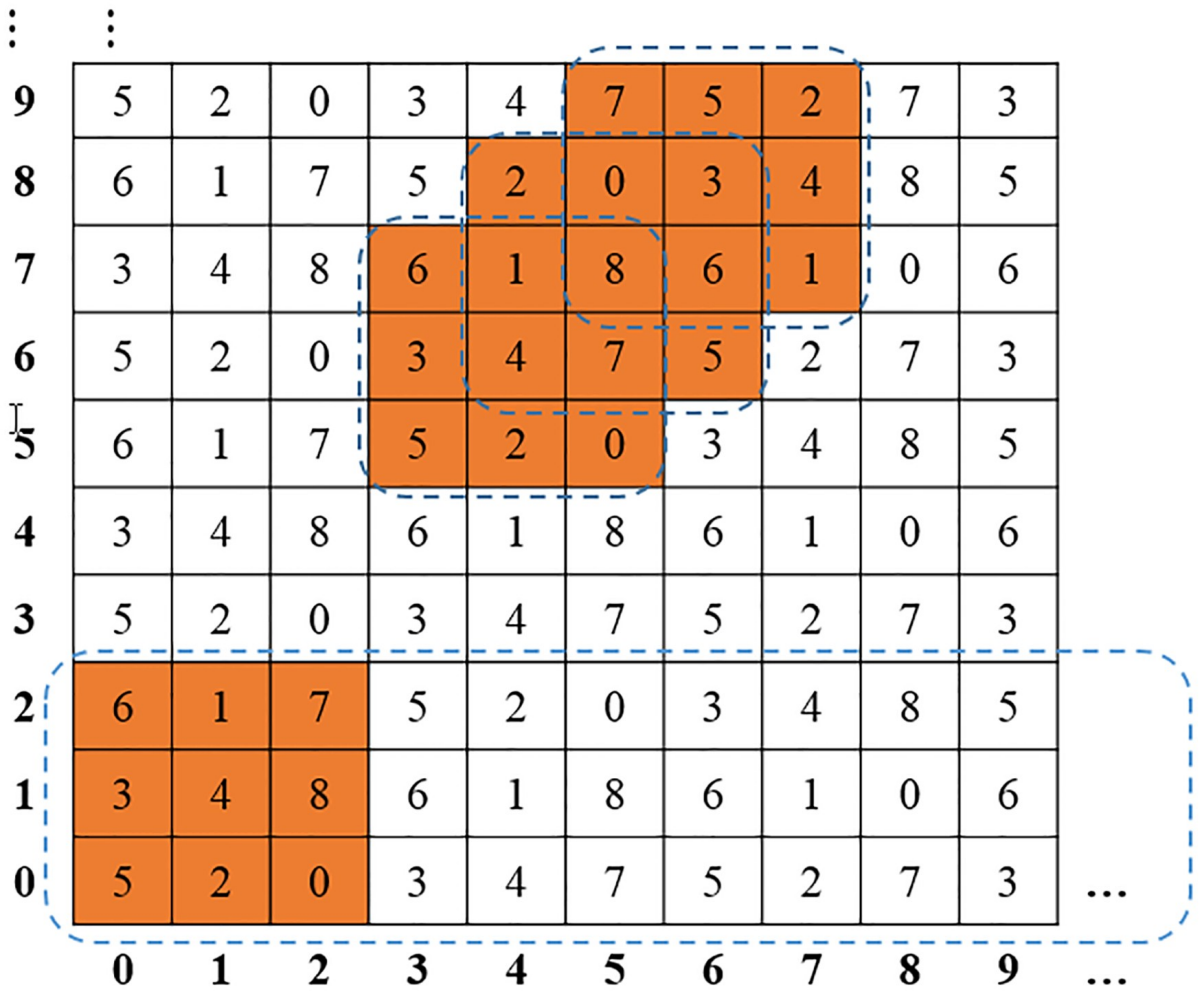
Example of a secure random matrix for *b* = 3.

Figs 2 and 3 illustrate the method of constructing a secure random matrix for *b* = 3 as an example. We can demonstrate in the Theorem 3 that there are at least $A_{2}^{2}$ different kinds of the initial matrices *m* in step 2, and each column of the rest matrices *M*″ has at least $A_{2}^{2}$ different solutions. The total number of generated reference matrices *M* are at least 10^78^ solutions, which is larger than the total number of the Sudoku for about 6.671 × 10^21^ [11].

### Embedding process

Before embedding the binary secret stream *E*, the parameter *b* must be determined. And the secure random matrix as shown in last subsection should be generated in advance. The detail embedding process is shown as follows.

Step 1 Let *I* be a cover image sized by *H* × *W*, where *H* and *W* are height and width of the image. For given *n* bits binary secret stream *E* = *e*_1_, *e*_2_, *e*_3_, ⋯, *e*_*n*_, we convert it into *N* secret digits *S* = *s*_1_, *s*_2_, *s*_3_, ⋯, *s*_*N*_ (*i*.*e*., *N* = ⌈*log*_*b*^2^_ *B*⌉) with the base-*b*^2^ numeral system, and the value of *N* is the total number of the converted secret that should not greater than *H* × *W*.
Step 2 According to the pre-constructed matrix *M* which was constructed in the last subsection, each cover pixel pair (*p*_*i*_, *p*_*i*+1_) could locate onto the reference matrix *M* at the *p*_*i*_th row and the *p*_*i*+1_th column as *M*(*p*_*i*_, *p*_*i*+1_).
Step 3 The element of *M*(*p*_*i*_, *p*_*i*+1_) and the *b* × *b* matrix of 8 numbers around it, the secret digit *s*_*i*_ can be found in this *b* × *b* matrix as element $M(p_{i}^{\prime },p_{i+1}^{\prime })$, and the original pixel pair *M*(*p*_*i*_, *p*_*i*+1_) is changed to $M(p_{i}^{\prime },p_{i+1}^{\prime })$.
Step 4 Repeat Step-2 and Step-3 until all secret information has been embedded completely. Finally, the stego image is set as $I^{\prime }=p_{1}^{\prime },p_{2}^{\prime },p_{3}^{\prime },\cdots ,p_{H\times W}$, which means that all the secret data is embedded.

For better understanding the embedding procedure, an example is taken as follows.

**Fig 4:**
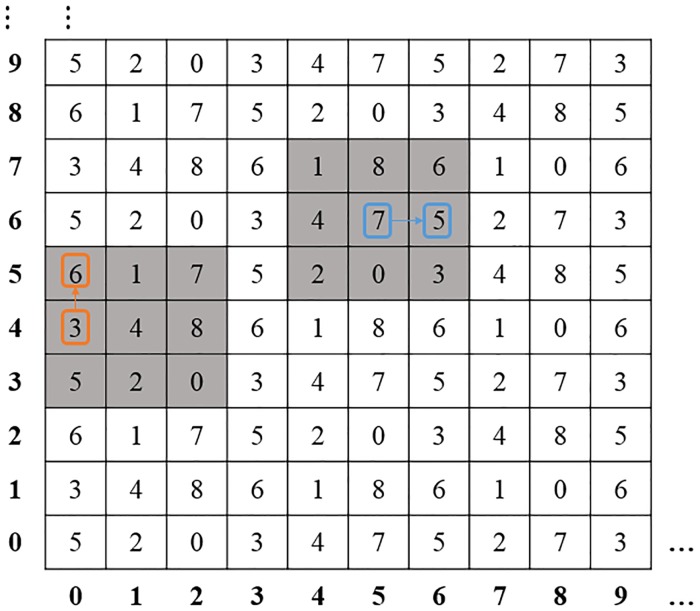
Example of the embedding process for *b* = 3.

Assume that the secret base-9 numeral digits (*s*_*i*_, *s*_*i*+1_) = (56)_9_(suppose *b* = 3), and two pixel pairs of the cover image are (5,6) (0,4). Then the process for embedding secret base-9 numeral system digits (*s*_*i*_, *s*_*i*+1_) into the pixel pairs is shown in Fig 4, which is described as follows.

1. Embed the secret digit 5 into (5, 6): Find the corresponding number *M*(5, 6) = 7 in the reference matrix M, and the matrix formed following Step 3. Then the element *M*(5, 7) is found as the selected element with secret 5. Finally, the original pixel pair (5, 6) in the cover image is changed into (5, 7).
2. Embed the secret digit 6 into (0, 4): Find the number *M*(0, 4) = 3 corresponding to element (0, 4) in the reference matrix *M*. It is easily to see that, although the position corresponding to (0, 4) is at the edge of the matrix *M*, the matrix could still be formed according to the Step 3. And the element *M*(5, 7) can also be found for the selected element with secret 6. Finally, the original pixel pair (0, 4) in the cover image is changed to (0, 5).

### Extracting process

The process of extract secret data from stego image is presented as following steps:

Step 1 Obtain the stego images and corresponding initial seed *k* of the PRNG and *b*, which are two important parameters to generate the reference matrix *M*.
Step 2 Divide the pixel values of the stego image into pixel pair that uses the same pairing technique.
Step 3 Generate the secure random matrix *M* in the embedding process by using *k* and *b*
Step 4 Find the number for $M(p_{i}^{\prime },p_{i+1}^{\prime })$ in the secure random matrix *M*. Obviously, the number indicates the embedding secret digit *s*_*i*_.
Step 5 After the stego pixel pairs are mapped in the reference matrix *M*, the original binary secret message *E* could exactly be retrieved from the extracted secret digits *S*.

## Results and discussion

**Fig 5:**
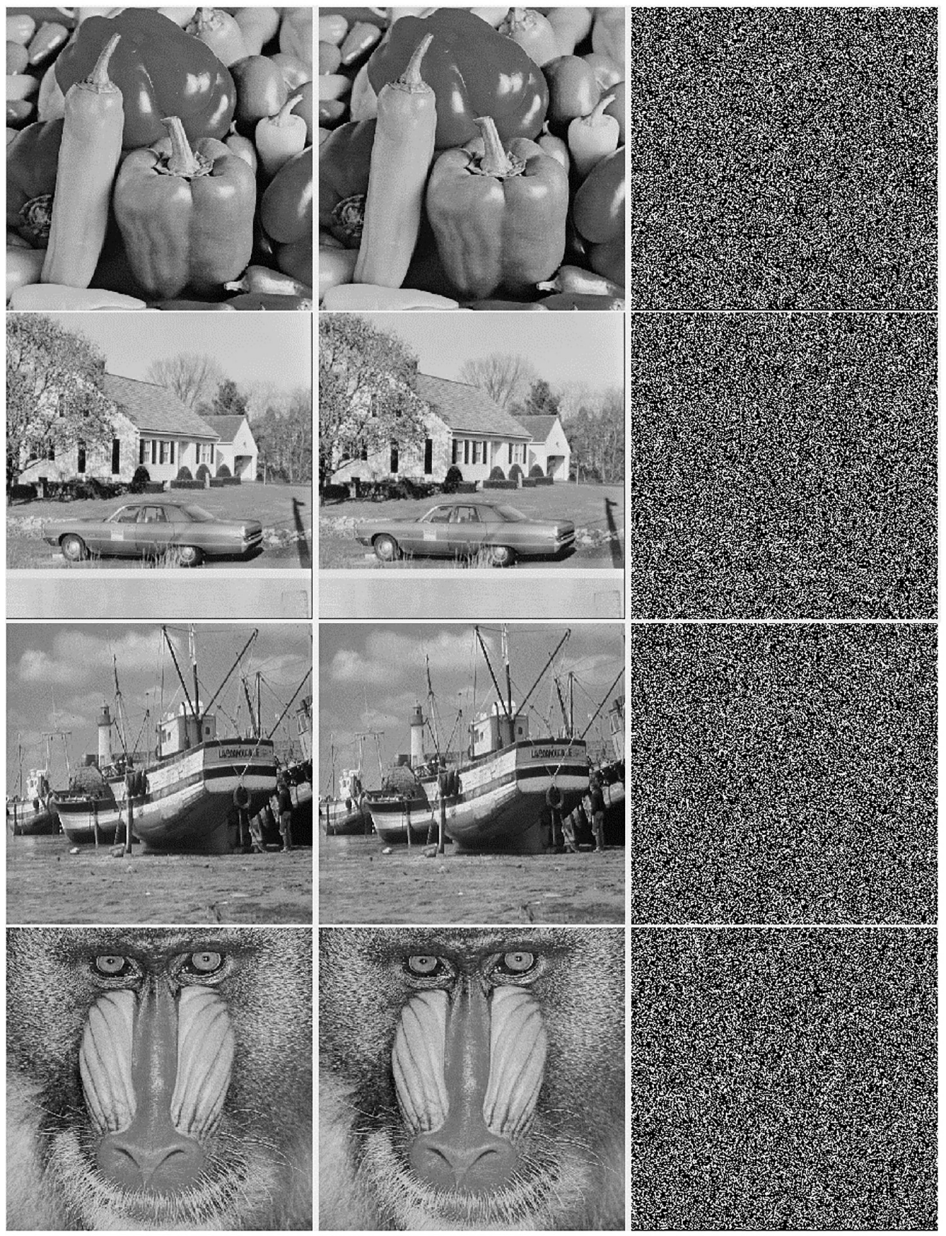
Part of standard images and the results after embedding secret data (left)standard image, (middle)stego image, (right)difference image.

In order to appraise the performance, we use standard image **Lena**, **Papers**, **Cameraman**, **House**, **Boat** and **Pirate** as test images which are shown in Fig 5. We notice that all the simulations of the experiments are implemented on an Intel (R) Core i5-3230 CPU@2.6 GHz and an-8-GB RAM through Python 3.5.

We consider two main parameters for evaluating the performance of image-based data-hiding schemes. The peak signal to noise ratio (PSNR) is applied in our simulations to evaluate the visual quality of stego images produced by the data hiding schemes. Actually, PSNR is a widely used and easy to implement the mechanism to evaluate the similarity between a cover image and a stego image. For the size of *H* × *W* cover image and stego image, the PSNR is formally defined as follows:

$$\begin{array}{c} \text{PSNR}=10{\text{log}}_{10}\frac{MAX^{2}}{\text{MSE}} \end{array}$$ (5)

where the *MAX* means the max value for each pixel, and the *mean square error* (*MSE*) denotes the value evaluating the difference between the original image and the stego image, which is formally is defined as follows:

$$\begin{array}{c} \text{MSE}=\frac{1}{H\times W}\sum_{i=1}^{H}\sum_{j=1}^{W}{\left(X_{ij}-Y_{ij}\right)}^{2} \end{array}$$ (6)

Actually, a large PSNR value means that the stego image has better visual quality (*i.e*., small distortion). On the contrary, a small PSNR value indicates that the stego image has poor visual quality which means that the image has a large distortion.

In terms of embedding capacity, we require that a stego image can carry secret data as much as possible. The embedding capacity is used as an indicator for the number of secret bits that can be embedded into a cover pixel. Informally, bit-per-pixel parameter *C* is applied to evaluate the embedding capacity and defined as follows.

$$\begin{array}{c} C=\frac{\left|S\right|}{H\times W} \end{array}$$ (7)

where |*S*| is the total number of secret bits that can be embedded into a cover image sized *H* × *W*. A large value of *C* indicates that a cover image can be used to hide a large number of secret bits required to be hidden.

**Fig 6:**
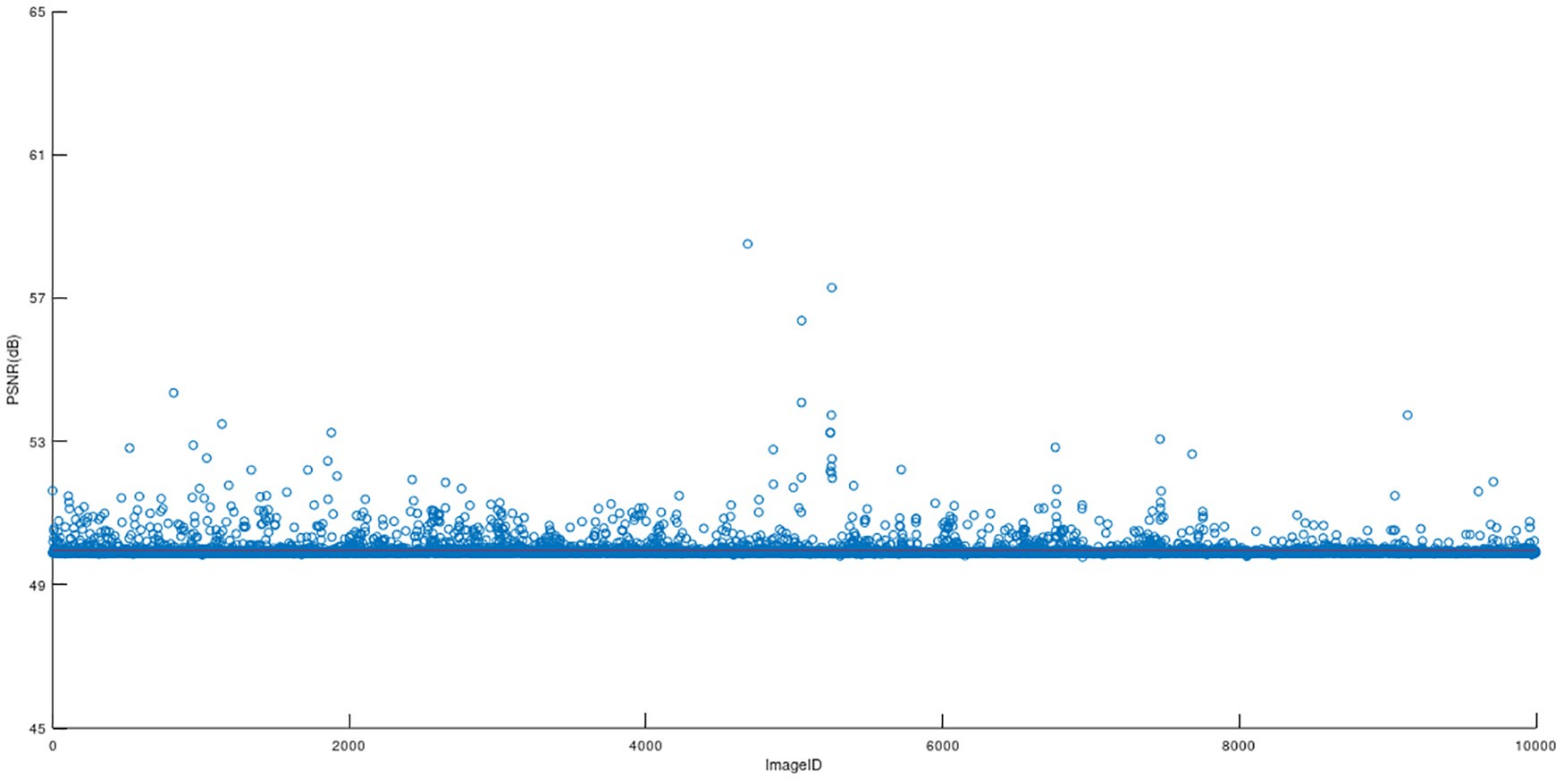
PSNR of the 10000 stego images from BOSSbase with *b* = 3.

In the Fig 6, every small circle presents a the PSNR value from 10000 stego images which are embedded with same secret data into 10000 greyscale image from BOSSbase, and the red line shows the average value of the 10000 stego images with full payload, which is 49.96 dB. It should be mentioned that a stego image have a higher PSNR than 30 dB will hard for human eyes to distinguish.

Table 1 shows the simulation results of Zhang-Wang’s method [10], Chang et al.’s method [11, 13] and the proposed method in terms of visual quality of stego images and embedding capacity. From the experimental results, the proposed approach can carry more secret data compared with Zhang-Wang’s method and Kim et al.’s [10]. Moreover, in the compact of image quality, the stego images generated by Kim et al.’s method and Chang et al.’s method have the same quality with ours. In general, our scheme has a higher embedding capacity under the condition of similar image quality.

**Table 1:** Comparison of proposed schemes and other schemes on PSNR and EC parameters for different images(for *b* = 3).

Images	[11]		[10]		[13]		Proposed scheme	
	PSNR	EC	PSNR	EC	PSNR	EC	PSNR	EC
Lena	44.97	1.5	50.86	1.37	49.42	1.5	49.95	1.5
Peppers	44.67	1.5	50.78	1.37	49.40	1.5	49.95	1.5
Baboon	44.68	1.5	50.69	1.37	49.39	1.5	49.96	1.5
Boat	44.94	1.5	50.64	1.37	49.40	1.5	49.95	1.5
Man	∖	∖	50.82	1.37	49.42	1.5	49.95	1.5
House	∖	∖	50.82	1.37	49.42	1.5	49.97	1.5

Table 2 shows the performance comparisons of PSNR among various reference table based schemes in the same embedding capacity 1.5 bpp(for *b* = 3 in proposed scheme). The experiments indicate that, PSNR in our scheme is 49.96, and [11] is 44.89, [19] is 45.37 and [20]is 48.67, respectively. Compared with related works, the proposed scheme achieves better PSNR than other schemes, more concretely, the PSNR of the proposed scheme provides more 5.07dB, 4.59dB and 1.29dB than the approaches in [11, 19, 20]. That is, our scheme has better visual quality with the same embedding capacity compared with others [11, 19, 20].

**Table 2:** Comparisons of PSNR between the proposed scheme and various reference-table-based schemes for *b* = 3.

Scheme	[11]	[19]	[20]	Proposed scheme
PSNR(EC = 1.5bpp)	44.89	45.37	48.67	**49.96**
Improved	**5.07**	**4.59**	**1.29**	∖

Table 3 shows the performance comparisons of PSNR among various reference table based schemes in the same embedding capacity 2 bpp(for *b* = 4 in proposed scheme). The experiments indicate that, the average of PSNR in our scheme is 46.37, and the classic Least Significant Bit(LSB) scheme is 45.32, [12] is 42.35 and [20] is 46.83, respectively. It can be find that compared with these works, the proposed scheme achieves better PSNR than the LSB scheme, the Chen et. al’s scheme [12] and similar with the Chang et. al’s scheme [6], which is means our scheme has better visual quality with the same embedding capacity compared with others [6, 12] for embedding capacity is 2 bpp(*b* = 4 for our scheme).

**Table 3:** PSNR comparison(payload = 2.00 bit for b = 4).

Images	LSB	[12]	[6]	Proposed scheme
Lena	45.33	43.03	46.83	46.38
Peppers	45.32	42.54	46.84	46.37
Baboon	45.32	41.41	46.84	46.37
Boat	45.33	42.17	46.83	46.38
Man	45.32	42.49	46.84	46.37
House	45.32	42.54	46.83	46.37
Average	45.32	42.35	46.83	46.37

Table 4 shows the comparisons of computational costs for embedding secret data. It is presented that the scheme of [11] has a loose set for searching the matching modified pixel pair, which requires three set for a search. And the scheme of [7] has a tight set for a search, but they need to solve a discrete optimization problem each embedding time and have to random the embedding sequence as the key for security, these cost a lot time. the schemes of [6, 14] are based on the same research for the turtle shell based reference matrix, and [6] makes it faster than [13] in embedding process through tightening the search range, but they also need to encrypt the secret data first for security. The LSB scheme has a high efficiency in embedding process, however, the security for the stego image is not considered. In our scheme, the only extra computation is generate a secure random matrix as reference matrix. And we have a tight set for searching the matching pixel pair, which embedding a base-*b*^2^ only need search in a set containing *b*^2^ numbers but no more additional computational costs.

**Table 4:** Comparison of computational costs.

Scheme	Encrypt secret data	Generate a reference matrix	embedding complexity	Search range	Additional computation
[11]	×	✔	easy	loose	×
[7]	×	×	complex	tight	random embedding sequence as key
[14]	✔	✔	easy	loose	×
[11, 19, 20]	✔	✔	easy	tight	×
LSB	✔	×	easy	no need	×
Proposed Scheme	×	✔	easy	tight	×

**Fig 7:**
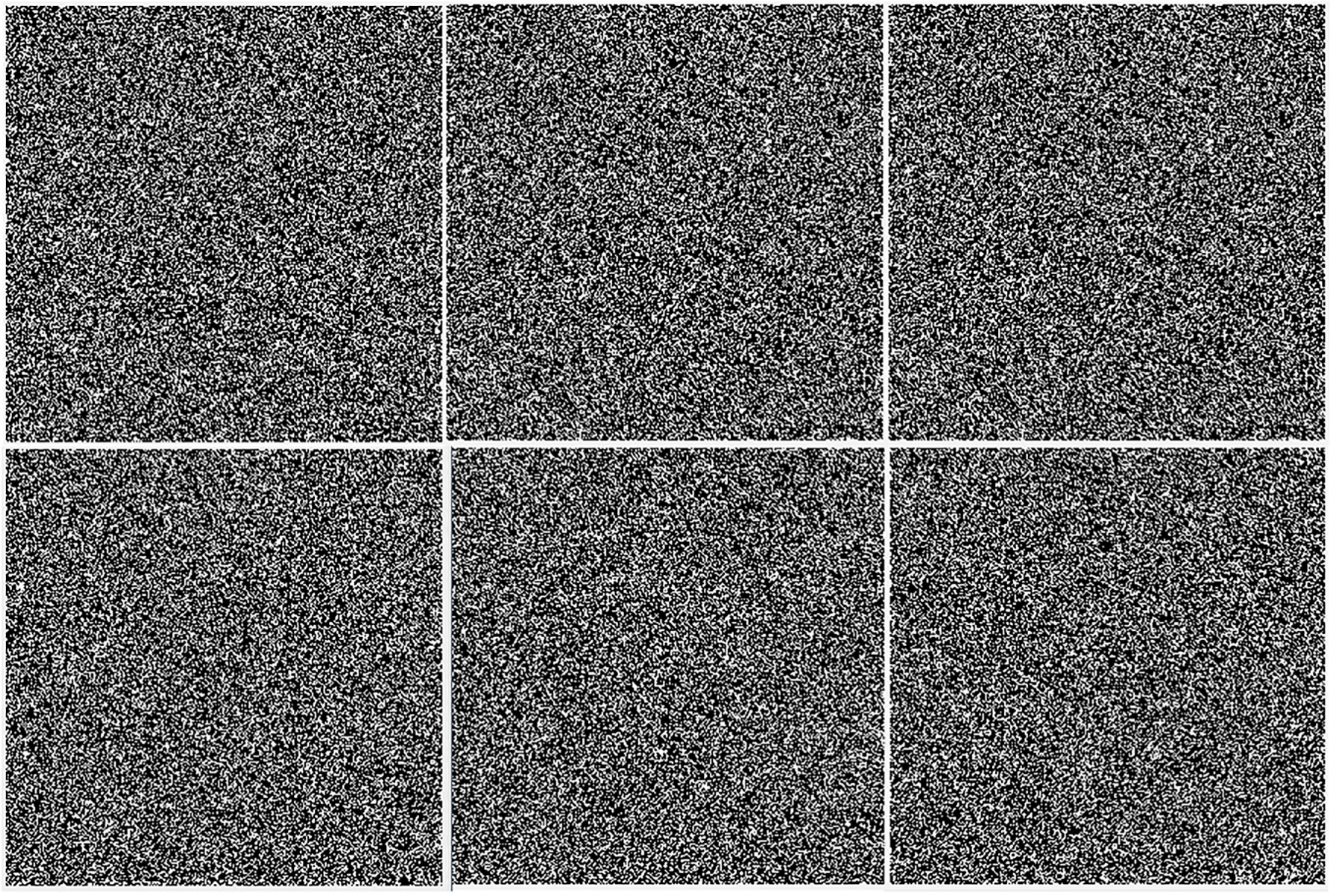
Difference images between the same cover image Lena and stego image with different key.

Fig 7 shows the difference image between the cover image and stego image for the same secret data with different key, the white blocks represent the pixel value of 255 and black blocks represent 0 or 1. These figures show the difference images between the cover image and the stego image where the standard image Lena is used as the cover image. From the experimental results, the proposed method can generate different stego images for the same cover image with embedded the same secret data. Once the secret key is changed, the stego image for the same cover image and secret data is also changed. So for anyone curious, without the secret key generated in embedding process, it’s hard to distinguish the difference between stego and cover images is useful data or just noise. After all, there are at least 10^7^8 kinds of secure random matrices, so we can produce at least 10^7^8 corresponding stego images for the same cover image.

We demonstrate in the Theorem 3 that there have 10^78^ kinds of possible matrices to be generated for the reference table in our scheme, which is hard for attackers to exploit exhaust attacks. And our scheme provides a high visual quality for the stego image to make sure that an unexpected user without the key can easily discover the secret message from the stego image. Thus the proposed approach provides an adaptive and high visual quality schemes for different situations.

## Conclusions

In this paper, we proposed an efficient and adaptive data hiding scheme by the coding on secure random matrix, in which two pixels are used to embed one secret digit according to the guidance of the reference matrix. The proposed scheme can be used in different situations for our adaptive data hiding scheme and high visual quality for stego image, and our method is secure because of a large number of possible solutions (about 10^78^ solutions) for resisting exhaust attacks, and only expected user who has the secret key *k* can be exactly extracted the secret from the stego image. Experimental results demonstrated that the proposed scheme is outperformed in both visual quality of stego image and efficiency.
